# Association between in-ICU red blood cells transfusion and 1-year mortality in ICU survivors

**DOI:** 10.1186/s13054-022-04171-1

**Published:** 2022-10-07

**Authors:** Alice Blet, Joel B. McNeil, Julie Josse, Bernard Cholley, Raphaël Cinotti, Gad Cotter, Agnès Dauvergne, Beth Davison, Kévin Duarte, Jacques Duranteau, Marie-Céline Fournier, Etienne Gayat, Samir Jaber, Sigismond Lasocki, Thomas Merkling, Katell Peoc’h, Imke Mayer, Malha Sadoune, Pierre-François Laterre, Romain Sonneville, Lorraine Ware, Alexandre Mebazaa, Antoine Kimmoun

**Affiliations:** 1 Université Paris Cité, Department of Anesthesiology, Critical Care and Burn Unit, INSERM, UMR-S 942, MASCOT, FCRIN INI-CRCT, Hôpitaux Universitaires Saint Louis – Lariboisière, Assistance Publique – Hôpitaux de Paris, Paris, France; 2 Division of Allergy, Pulmonary, and Critical Care Medicine, Department of Medicine, University School of Medicine, Nashville, Vanderbilt, TN USA; 3 Université de Montpellier, IDESP-Institut Desbrest d’Épidémiologie et de Santé Publique, PREMEDICAL - Médecine de Précision Par Intégration de Données et Inférence Causale, CRISAM- Inria Sophia Antipolis – Méditerranée, Montpellier, France; 4 Université Paris Cité, INSERM UMR_S 1140 “Innovations Thérapeutiques en Hémostase”, 75006 Paris, France; 5 Hôpital Européen Georges Pompidou, AP-HP, 75015 Paris, France; 6 University of Nantes, Department of Anesthesia and Critical Care, Hôtel Dieu, Intensive Care Unit, University Hospital of Nantes, Nantes, France; 7 Momentum Research, Inc., Chapel Hill, NC 27517 USA; 8 Université Paris Cité, Department of Biochemistry, Assistance Publique – Hôpitaux de Paris, Hôpital Beaujon, Clichy, France; 9 Université de Lorraine, INSERM 1433 CIC-P CHRU de Nancy, Inserm U1116 and FCRIN INI-CRCT, Nancy, France; 10 Université Paris-Sud, Anesthesia and Intensive Care Department, Assistance Publique Hôpitaux de Paris, Hôpital de Bicêtre, Le Kremlin-Bicêtre, France; 11 Université de Montpellier, Department of Anesthesia and Intensive Care Unit, PhyMedExp, INSERM U1046, CNRS UMR, 9214, CHRU de Montpellier, Hôpital Saint Eloi, Montpellier, France; 12 Université d’Angers, Department of Anesthesia and Intensive Care Unit, CHU d’Angers, Angers, France; 13 Université Paris Cité, Department of Biochemistry, CRI INSERM UMR1149, HUPNVS, Assistance Publique – Hôpitaux de Paris, Paris, France; 14 Institute for Public Health, Charité – Universitätsmedizin Berlin, Berlin, Germany; 15 Intensive Care Unit, Clinique Universitaire St Luc UCL, Brussels, Belgium; 16 Université Paris Cité, Department of Intensive Care Medicine, INSERM UMR1148, HUPNVS, Assistance Publique – Hôpitaux de Paris, Paris, France; 17 Université de Lorraine, CHRU de Nancy, Intensive Care Medicine Babois, INSERM U1116, FCRIN INI-CRCT, Nancy, France

**Keywords:** Transfusion, Mortality, Critical care unit

## Abstract

**Background:**

Impact of in-ICU transfusion on long-term outcomes remains unknown. The purpose of this study was to assess in critical-care survivors the association between in-ICU red blood cells transfusion and 1-year mortality.

**Methods:**

FROG-ICU, a multicenter European study enrolling all-comers critical care patients was analyzed (*n* = 1551). Association between red blood cells transfusion administered in intensive care unit and 1-year mortality in critical care survivors was analyzed using an augmented inverse probability of treatment weighting-augmented inverse probability of censoring weighting method to control confounders.

**Results:**

Among the 1551 ICU-survivors, 42% received at least one unit of red blood cells while in intensive care unit. Patients in the transfusion group had greater severity scores than those in the no-transfusion group. According to unweighted analysis, 1-year post-critical care mortality was greater in the transfusion group compared to the no-transfusion group (hazard ratio (HR) 1.78, 95% CI 1.45–2.16). Weighted analyses including 40 confounders, showed that transfusion remained associated with a higher risk of long-term mortality (HR 1.21, 95% CI 1.06–1.46).

**Conclusions:**

Our results suggest a high incidence of in-ICU RBC transfusion and that in-ICU transfusion is associated with a higher 1-year mortality among in-ICU survivors.

*Trial registration* (NCT01367093; Registered 6 June 2011).

**Graphic Abstract:**

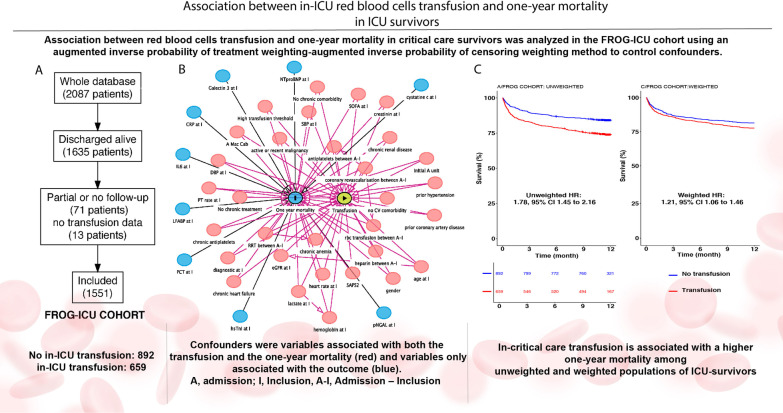

**Supplementary Information:**

The online version contains supplementary material available at 10.1186/s13054-022-04171-1.

## Introduction

Blood transfusion is one of the most common procedures performed during hospitalization, especially in intensive care units (ICU). Patients received, appropriately or not, a transfusion of RBC alone or, in some cases, combined with fresh frozen plasma and/or platelets. Data on transfusion in critical care patients are scarce, declarative, and studies have focused only on short-term mortality [1, 2]. Thus, Vincent et al. evidenced, already two decades ago, in a prospective European cohort of 3534 patients in ICU, an association between transfusion and both organ dysfunction and short-term mortality [1]. The transfusion of RBC, when properly indicated, yields short-term benefits, including an increase in oxygen delivery to tissues. By contrast, RBC transfusion, especially when it is unduly prescribed, could be also associated with short-term harm, including volume overload, transfusion-related acute lung injury, infections, hemolysis, or adverse immunomodulation [3]. Apart from these quite rare adverse events, transfusion could be associated, in relation or not with its storage, with a faster transfusion clearance resulting in hemolysis and systemic inflammation and ultimately with a possible higher risk of kidney dysfunction [4–7]. Thus, to avoid these harmful effects, a restrictive approach toward transfusion practice has been advocated and shown to be non-inferior to the liberal strategy, and it is now recommended by practice guidelines [8–12]. Recommended transfusion thresholds are a hemoglobin concentration [Hb] < 7 g/dL for stable non-bleeding patients, and [Hb] < 8 g/dL in patients with coexisting cardiovascular disease and those undergoing cardiac or orthopedic surgery [13]. Restrictive transfusion strategies have been shown to improve short-term outcomes in several patient populations, including cardiac surgery [14, 15]. Conversely, the long-term impact of various transfusion strategies remains unknown. Furthermore, whether a highly restrictive strategy is associated with benefits or harm during the months following an ICU stay remains unknown. To test this hypothesis, a randomised controlled study would be the recommended approach, achieving similar groups and thus a straight assessment of the effects of transfusion on the long-term outcome. However, such a randomised controlled study would be difficult to perform since transfusion may be considered by many investigators as essential for the potential survival of some critically ill patients. The use of a cohort study raises other issues. Due to differences in patient characteristics affecting the decision to treat, a direct association between transfusion and long-term outcome might result in a biased estimation. Propensity score matching may help to overcome this issue by minimising potential biases. However, this approach has three well-known major shortcomings [16]. (1) unmeasured or persisting confounders may result in a biased association. (2) variables associated to the outcome but not to the treatment are not included in the analysis. (3) many patients are excluded when a matching procedure is performed, restricting the generalisability of the results. To overcome these limitations, we performed an augmented inverse propensity weighted statistical method that is based on a propensity score, but no patient is excluded and a specific regression model focused on outcome-associated variables is added to account patient’s severity unrelated to the transfusion [17].

The purpose of this study was to assess the association, first, between RBC transfusion during the ICU stay and long-term survival after ICU discharge and, second, between transfusion, hemolysis, and kidney function.

## Methods

### Study design and Patients

We analysed data from the French and European Outcome Registry in Intensive Care Units (FROG-ICU). The FROG-ICU study (www.clinicaltrials.gov/show/NCT01367093) was a prospective, observational, multicenter cohort study, designed to assess all-cause 1-year mortality after ICU discharge and to identify the mortality risk factors during the year following discharge from the ICU [18]. The study protocol has been previously published [19]. Briefly, the study was conducted in France and in Belgium and was approved by ethical committees of both countries [20]. The study involved ICUs of 21 centers. The study cohort included 2087 consecutive patients, who were admitted to the ICU in any of the participating centers from August 2011–June 2013 when the following inclusion criteria were met: invasive mechanical ventilation support for at least 24 h and/or treatment with a vasoactive agent (norepinephrine, epinephrine, dobutamine, levosimendan, phosphodiesterase inhibitors) for more than 24 h. Non-inclusion keys criteria were: < 18 years old, severe brain injury or brain death or a persistent vegetative state, pregnancy or breastfeeding, transplantation in the past 12 months, not expected to survive or to leave the hospital and/or no social security coverage [18].

### Data collection and biological samples

In the FROG-ICU study, the following patient data were collected at the time of inclusion: demographics, past medical history, measure of premorbid status (Mac Cabe score classifies all hospitalised patients into 3 categories: (1) non-fatal disease, (2) fatal disease within 5 year and (3) fatal disease within 1 year), ICU admission diagnosis, hemodynamic, and severity of disease classification scores. The need for organ support (vasopressors, renal replacement therapy) and the number of transfused units (packed RBC, fresh frozen plasma, or platelets) were recorded throughout the ICU stay. In addition, critical parameters and clinical events between admission and inclusion were also recorded (*e.g.*transfusion status, SAPS 2, antiplatelets treatments, coronary revascularization, heparin treatment…). Biological routine parameters were collected at inclusion and at discharge to study risk factors associated with 1-year survival. Hemoglobin concentration was measured daily from inclusion to day 3, and then bi-weekly until discharge or death. A biobank was created and stored at − 80 °C with blood samples collected within 24 h after patient inclusion and at discharge. Among the 1551 patients discharged alive from ICU and who were included in this study, the following biomarkers were centrally measured a posteriori (1) plasma levels of hs troponin I (Abbott, Abbott Park, IL, USA), N-Terminal pro-Brain Natriuretic Peptide (NT-proBNP, Roche Diagnostics GmbH, Mannheim, Germany), proenkephalin A 119–159 (penKid, Sphingotec GmbH, Hennigsdorf, Germany), neutrophil gelatinase associated lipocalin (NGAL), galectin-3 (Abbott, Abbott Park, IL, USA), haptoglobin (Architect, Abbott Park, IL, USA), interleukin 6 (IL-6, Elecsys, Roche, Penzberg Germany) and (2) urine concentrations of: NGAL (Abbott, Abbott Park, IL, USA), cystacin C (Abbott, Abbott Park, IL, USA), liver fatty acid binding protein (L-FABP, Nordia L-FABP; Sekisui Medical Co., Ltd., Tokyo, Japan). To account the close relationship between haptoglobin expression and IL-6 levels, haptoglobin level was normalised on IL-6 level at discharge time point [21].

### Objectives

The primary objective was to describe the association between in-ICU RBC transfusion and 1-year mortality after ICU discharge. Exploratory analyses were also conducted to determine the discharge factors associated with 1-year post-ICU mortality, specifically hemolysis and kidney injury.

### Statistical methods

Additional file 1: Figure S1 summarises the statistical analysis performed.

Patients were separated into two groups: those who received RBC transfusion (*i.e.*: at least one unit of packed RBC) during their ICU stay, and those who did not. Survival was observed over a period of 1 year following ICU discharge.

Of note, to assess the impact of transfusion in patient selection at discharge, 1-year survival curves were additionally also drawn from admission to ICU with the whole FROG cohort population.

#### Primary outcomes

The primary outcome was 1-year survival after ICU discharge. The average treatment effect of RBC transfusion on survival was estimated from the survival curves of patients with and without RBC transfusion, from the associated hazard ratio, and from the differences in restricted mean survival times (RMST). The latter corresponds to the average number of days gained or lost in terms of 1-year overall survival after ICU discharge between patients transfused and not transfused during their ICU stay. The confidence intervals associated with these estimated values were computed from 100 bootstrap samples.

#### Data description

Data were expressed as median (inter-quartile range, IQR), mean ± standard deviation (SD), or number (percentage). Numerical data were compared using t-test or Wilcoxon rank test, while categorical variables were compared using χ^2^ or Fischer’s test, as appropriate. Repeated measures of continuous variables were handled by a linear mixed model tested with Kenward-Roger’s F tests.

#### Management of missing data

Two approaches were used for handling the missing values: a parametric one with multiple imputations by chained equations (MICE), and a non-parametric one with random forest-missingness incorporated in attributes (MIA). Details of the two methods are provided in the Additional file 2. Number of missing values per variable was also added in Additional file 1: Figure S2.

#### Models

- Semi-parametric and non-parametric approaches to estimate models.

Two different approaches were considered to estimate the effect of RBC transfusion on 1-year mortality: a semi-parametric approach and a non-parametric approach. In the semi-parametric approach, we used Cox models to model the survival and the censoring. Treatment allocation was modelled with a propensity score calculated from a logistic regression. In the non-parametric approach, we modelled with random survival forests the survival, the censoring, and the treatment allocation.

Under these semi-parametric and non-parametric approaches, two estimators (see estimators performed below) were applied to assess the study’s primary outcomes based on models in which identification of confounding factors was required.

- Confounding variables selection.

A three round Delphi method including experts in critical care and transfusion was used to identify the confounding variables necessary to build the different models. Additional file 1: Figure S3 shows the causal inference diagram applied in a directed acyclic graph, differentiating variables assessed as predictors of the outcome but unrelated to the treatment assignment and the variables assessed as predictors of both treatment and outcome.

#### Causal inference estimators

- Estimator performed to draw survival curves.

The unweighted curves were estimated with the Kaplan Meier estimator, the unweighted hazard ratio was estimated with a Cox regression with only the transfusion status as a variable. The weighted survival curves were built with the non-parametric doubly robust estimator, *i.e.:* augmented inverse probability of treatment weighting—augmented inverse probability of censoring weighting (AIPTW-AIPCW using survival and random forests-MIA method for management of missing values, see Additional file 2). The weighted hazard ratio was computed from the weighted survival curves by averaging the hazard ratio at each time point. This estimator was calculated in our main population of interest: ICU-survivors from ICU-discharge but also, to ensure that the result was not driven by patient’s ICU stay, in the whole initial population (including ICU-survivors and non-survivors) from ICU admission.

- Estimators performed.

The three following causal inference estimators were performed to calculate the RMST: (1) the unweighted estimation with no adjustment, then, in parametric and non-parametric approaches of missing values: (2) the inverse probability of treatment weighting with the Kaplan Meier estimator (IPTW), (3) the AIPTW-AIPCW. Details of each estimator are provided in the Additional file 2.

#### Exploratory analysis

Except for the packed red blood cells unit number threshold associated with 1-year mortality (see below), all exploratory analyses were performed using parametric (with MICE) imputed FROG-ICU cohort.

- Packed red blood cells threshold.

With non-imputed data, we looked for the number of the packed RBC units for which there was a maximal increase in 1-year mortality after ICU discharge. First, the log linearity assumption was checked using the restricted cubic spline method. Given the lack of log linearity, the number of transfused packed RBC units has been dichotomized according to an optimal level determined using the most significant *p* value from the log rank test. Subsequently, this threshold has been validated using a univariate Cox model.

A two-tailed *p* value of less than 0.05 was considered significant. Statistical analyses were performed using R v3.6.3 (R Foundation for Statistical Computing, Vienna, Austria).

## Results

### Characteristics of FROG-ICU cohort

Of the 2087 ICU patients who consented to participate in the FROG-ICU study, 1635 were discharged alive from the ICU. Among them, 84 patients were excluded from the current study due to missing data on transfusion status or follow-up (Additional file 1: Flow chart, Figure E4), leaving 1551 ICU survivors in our cohort.

The main reasons for ICU admission are presented in detail in Table 1. On admission, SAPS-II score was 46 (34–60) and Charlson’s score was 1 (0–2). The all-cause mortality at 1 year after ICU discharge for these patients was 20%. Within the cohort, 659 (42%) patients received at least one unit of RBC transfusion during their ICU stay, and 248 (16%) patients received a combination of RBC transfusion and platelets and/or plasma transfusion. Patient characteristics including demographics, comorbidities, need for a renal replacement therapy and autologous transfusion as well as outcomes (length of ICU stay and 1-year mortality) are presented in Table 1. The median number of RBC transfused was 4 (2–7) and 49 patients received only one RBC unit. The first transfusion was administered at 2 (1–6) days following ICU admission (Additional file 1: Figure S5). Additional file 1: Figure S6 shows the time-course of hemoglobin concentration for transfused and non-transfused patients over the ICU stay.

**Table 1:** Characteristics of patients discharged alive in the FROG-ICU cohort

Variables	*n*	Global median (Q1–Q3) or *n* (%)	*n*	No in-ICU transfusion median (Q1–Q3) or *n* (%)	*n*	In-ICU Transfusion median (Q1–Q3) or *n* (%)	*p* value
*Demographic*							
Age (years)	1551	61 (49–73)	892	60 (48–71)	659	63 (51–74)	0.0003
Female gender	1551	566 (36%)	892	314 (35%)	659	252 (38%)	0.22
*Medical history*							
Charlson score	1551	1 (0–2)	892	0 (0–2)	659	1 (0–2)	< 0.0001
Hypertension	1550	634 (41%)	892	337 (38%)	658	297 (45%)	0.004
Coronary artery disease	1550	123 (8%)	892	49 (5%)	658	74 (11%)	< 0.0001
Chronic heart failure	1550	106 (7%)	892	54 (6%)	658	52 (8%)	0.15
Diabetes mellitus	1550	271 (17%)	892	143 (16%)	658	128 (19%)	0.080
Chronic obstructive pulmonary disease	1550	171 (11%)	892	102 (11%)	658	69 (10%)	0.56
Chronic renal disease	1550	162 (10%)	892	58 (7%)	658	104 (16%)	< 0.0001
Chronic liver disease	1550	98 (6%)	892	49 (5%)	658	49 (7%)	0.12
Active or recent malignant disease	1550	186 (12%)	892	77 (9%)	658	109 (17%)	< 0.0001
*Causes for admission*	1551		892		659		< 0.0001
Cardiac causes of admission		221 (14%)		150 (17%)		71 (11%)	
Hemorrhagic shock*		83 (5%)		10 (1%)		73 (11%)	
Acute respiratory failure		299 (19%)		204 (23%)		95 (14%)	
Neurologic causes of admission		240 (15%)		200 (22%)		40 (6%)	
Others		96 (6%)		45 (5%)		51 (8%)	
Polytrauma		86 (6%)		38 (4%)		48 (7%)	
Post-surgery		156 (10%)		73 (8%)		83 (13%)	
Sepsis		370 (24%)		172 (19%)		198 (30%)	
*Severity scores*							
SAPS II score	1550	46 (34–60)	891	46 (34–59)	659	46 (35–61)	0.085
SOFA score	1146	7 (4–10)	633	7 (4–10)	513	8 (5–11)	< 0.0001
* Management*							
Renal replacement therapy	1551	283 (18%)	892	102 (11%)	659	181 (27%)	< 0.0001
Red blood cell transfusion	1551	659 (42%)	892	0 (0%)	659	659 (100%)	< 0.0001
Platelets transfusion	1551	223 (14%)	892	20 (2%)	659	203 (31%)	< 0.0001
Number of red blood transfusions	1551	0 (0–3)	892	0 (0–0)	659	4 (2–7)	< 0.0001
*Outcomes*							
Length of stay in ICU (days)	1551	12 (7–21)	892	10 (6–16)	659	16 (9–27)	< 0.0001
One-year mortality	1551	312 (20%)	892	141 (16%)	659	171 (26%)	< 0.0001

*Hemorrhagic shock patients receiving transfusion before admission in ICU but not in ICU or between admission an inclusion in the FROG-ICU study

### Primary outcomes: Transfusion and outcome in ICU survivors

**Fig. 1:**
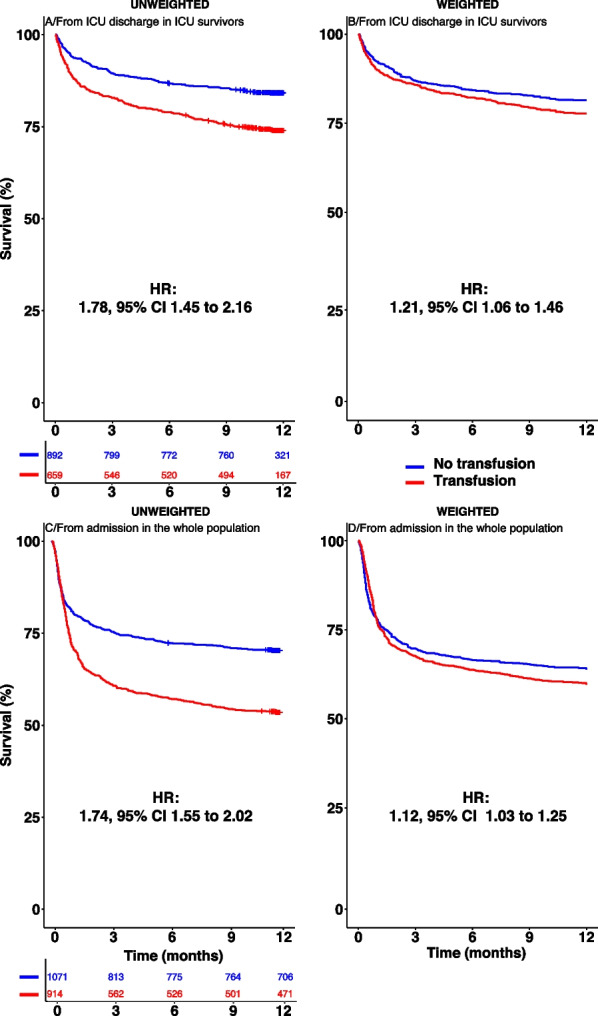
One-year survival according to transfusion status from discharge in unweighted (**A**) and weighted (**B**) populations and from admission in the whole population in unweighted (**C**) and weighted (**D**) populations. Weighting has been performed with an AIPTW-AIPCW estimator from the random forest-MIA (non-parametric) imputation. For the whole population sample size from 2087 patients, 72 patients had no or partial follow up and 30 no transfusion data resulting in 1071 patients in the no transfusion group and 914 patients in the transfusion group

**Fig. 2:**
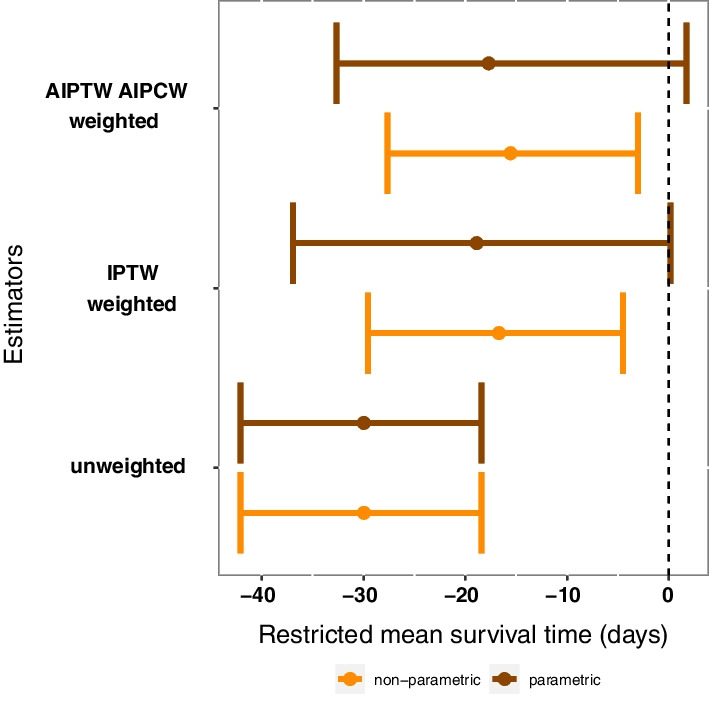
Averaged effect of transfusion on the restricted survival time for the first 365 days after discharge estimated from non-parametric and parametric methods of missing values management. IPTW: inverse probability of treatment weighting, AIPTW-AIPCW: augmented inverse probability treatment weighting-augmented inverse probability censoring weighting

Figure 1 panel A shows the unweighted 1-year post-ICU survival in the FROG-ICU cohort from ICU discharge in ICU-survivors, in the transfusion group compared to the non-transfusion group (Hazard ratio (HR) 1.78, 95% CI 1.45–2.16). Distribution of propensity scores estimating regions of common support were calculated from the parametric approach and the non-parametric approach (Additional file 1: Figure S7, panel A and B). Additional file 1: Figure S8 shows the standardized mean differences between transfusion and no transfusion groups in non-weighted and propensity score-weighted populations. After weighting of confounding variables using AIPTW-AIPCW estimator, the risk of death remained higher in the transfusion group (HR 1.21, 95% CI 1.06–1.46, Fig. 1 panel B). Moreover, RMST was consistently reduced in the transfusion group whatever the estimators used with both parametric and non-parametric approaches of missing values management (Fig. 2). For instance, in the transfusion group compared to the non- transfusion group, the RMST was − 30, 95% CI − 42 to − 18 days in the non-weighted cohort and the RMST, calculated from the non-parametric approach, was -16, 95% CI − 28 to − 3 days after the AIPTW-AIPCW estimation. As a sensitivity analysis, Fig. 1 also shows the unweighted (panel C) and weighted (panel D) 1-year survival from admission in the transfusion group and in the non-transfusion group in the whole population. Curves were superimposed in the two panels during the first month.

### Exploratory analysis in the FROG cohort

According to the sensitivity analysis, the interaction between chronic kidney function and 1-year ICU mortality in patients who received transfusion was the only significant clinical variable (*p* = 0.0008, Additional file 1: Figure S9). Plasmatic and urinary renal biomarkers of acute kidney injury (plasmatic and urinary N-GAL, urinary L-FABP, plasmatic Penkid) measured at discharge were all increased in the transfusion group compared to the no transfusion group (see Additional file 1: Table S1). Regarding plasmatic cardiac biomarkers measured at discharge, high sensitivity troponin I was similar between transfusion and no transfusion groups while galectin-3 and NT-pro BNP were both increased in the transfusion group (Additional file 1: Table S2). Except for plasma N-GAL, no interaction was found between biomarkers and in-ICU transfusion on 1-year mortality (Additional file 1: Figure S10). Additional file 1: Figure S11 panel A further shows that, at discharge, patients in the transfusion group had a lower plasma haptoglobin/IL-6 ratio. Additional file 1: Figure S11 panel B also shows that the lower the haptoglobin/IL-6 ratio the lower creatinine clearance at discharge (*p* < 0.0001).

Finally, the association between transfusion and 1-year mortality appeared to be significant from the very first RBC unit prescribed (see Additional file 1: Figure S12).

## Discussion

In our large cohort of ICU survivors, we observed a high incidence of in-ICU RBC transfusion and a higher risk of 1-year mortality in patients who received RBC transfusion during their ICU stay. Few studies have compared outcomes between transfused and non-transfused critically ill patients and most often for short-term mortality. Vincent et al. compared in an international cohort study the effects of RBC transfusion on hospital mortality. They found, in patients receiving RBC transfusion, a slight reduced risk of in-hospital mortality in most severely ill patients and lowest admission hemoglobin levels. This result is not inconsistent with our, while being difficult to compare as we only studied the effect of in-ICU transfusions in ICU-survivors on 1-year outcome [2]. Pattakos et al. analysed patients undergoing cardiac surgery who refused transfusion (Jehovah’s Witnesses) and compared them to matched patients who received transfusions. They observed a better 1-year survival and similar 20-year survival in patients who did not receive transfusion [22]. In critically ill patients, studies have only compared two strategies of transfusion—liberal *versus* restrictive—associated with different pre-transfusion thresholds of hemoglobin, on short-term outcomes [14, 23]. These studies, including exclusively patients requiring a transfusion, recommended a restrictive strategy. We observed that most transfused patients had a nadir of hemoglobin that was above than the recommended guideline thresholds of 7 or 8 g/dL [8, 11, 12]. Several observational studies also reported pre-transfusion hemoglobin concentrations that were higher than the recommended guideline thresholds [2, 24]. Accordingly, the high incidence of transfusion in our cohort and in other cohorts suggests that patient blood management programs need to be more aggressively implemented in ICUs since the suggested detrimental effects on 1-year survival appears upon the very first packed RBC. Nevertheless, transfusion alone is not accountable for the progressive survival decline observed in both groups. One-year mortality is also affected by the events occurring the ICU stay: RRT, ICU-acquired weakness. An effective blood management program should also be integrated in a more global program to reduce the burden of ICU.

Missing data and unmeasured confounders will always remain significant limitations to causal inference approaches applied on cohort studies. To handle missing data, we applied both parametric and non-parametric methods to manage missing values according to the best actual standards. While results were similar with these two methods, the non-parametric method (random forest- MIA method) seemed to provide more accuracy with less variability compared to other estimators. Compared to usual propensity-derived techniques, AIPTW-AIPCW estimation included not only variables that were associated to treatment and outcome, but also variables only associated with outcome. This estimator also has a “doubly robust” property, meaning that to be asymptotically unbiased, only one of the two model needs to be properly specified [25]. Taken as a whole, this approach gives a better estimate of transfusion effect than an estimation based on a matched cohort from a propensity score.

Since all patients were discharged from ICU alive, we hypothesised that some organs, namely the heart or the kidney, might remain injured in the transfusion group after ICU discharge. Our results indicate that alterations in cardiovascular biomarkers at discharge were slightly more pronounced in patients who received transfusion versus those who did not. By contrast, eGFR and all studied plasma and urinary markers of renal function were markedly altered in the transfusion group. The relationship between altered kidney function at discharge and the poor long-term post-ICU outcome has already been described [26]. Accordingly, the observed increase in 1-year mortality in the transfusion group could potentially be related to clinical and/or subclinical persistent acute kidney injury. The mechanism responsible for the worse kidney function in the transfusion group is unknown. However, we found lower circulating haptoglobin concentrations at discharge, suggesting that hemolysis is possibly more frequent in transfused patients during their ICU stay. Intravascular hemolysis has consistently been proposed as a critical modulator of vascular function after RBC transfusion [27, 28]. In many settings, including critical illness, hemolysis has been associated with worsening renal function and death [29].

Our study has several limitations. First, it is an observational retrospective study with all inherent biases to this design. Thus, despite a complex weighting method including 40 variables chosen a priori, uncontrolled confounders may persist. Our population of interest consisted in ICU survivors transfused during their ICU stay, and for whom follow-up started after ICU discharge. By only selecting ICU-survivors, a selection bias might have altered the true effect of transfusion on 1-year mortality. However, the association between transfusion and one year mortality was also found in the whole population at admission. Second, this cohort was not specifically designed to study transfusion and some important variables were not comprehensively recorded such as the delay between inclusion and first transfusion in ICU or the hemoglobin level before an RBC transfusion. Third, we also might have misclassified some patients who were transfused and were included in the no-transfusion group. Indeed, we did not consider potential transfusions administered before ICU admission (ward, operating room, or emergency department) or following ICU discharge as demonstrated by 10 hemorrhagic shocks in the no in-ICU transfusion group who received RBC transfusion before ICU admission and thus before the inclusion. However, except for patients in the operating room with acute hemorrhage and stabilised with transfusion and surgery, failing to recognise patients that received transfusion before ICU would most probably result in less difference between the groups. Of note, transfusion is much less frequent in other wards than in ICU [30]. Fourth, two distinct transfusion profiles were found: patients urgently requiring transfusion upon admission and patients requiring non-urgent transfusion, most often lately in the ICU-stay. Inclusion of these two distinct profiles might be considered inadequate. However, the sensitivity analysis found that the long-term impact of in-ICU transfusion was similar for all causes of admission including a subgroup including polytrauma and hemorrhagic shock patients. For the latter, we still don’t precisely know the impact of high in-ICU transfusion doses on the long-term outcome as too few patients received more than 3 RBC transfusion. Fifth, although we have used the most advanced statistical methods to input missing data and have used a large cohort to verify the robustness of our findings, the timing of transfusion was highly variable among patients. Thus, some confounding variables were inherently uncontrolled. Sixth, the impacts on 1-year survival of transfusion dose and/or co-transfusions (associating RBC with platelet concentrates and/or fresh frozen plasma concentrates) were not evaluated in this study.

Finally, characteristics of RBC, especially duration of storage and gender of RBC donors were unknown, preventing us to assess their previously reported effect in our cohorts [31–33].

In summary, analyses of a large ICU cohort from Europe suggested a high incidence of in-ICU RBC transfusion and that transfusion during the ICU stay was associated with a higher risk of death during the year following discharge. Our results suggest that, at bedside, intensivists should pursue their efforts to restrict red blood cell transfusion to patients who really need it.

Additional prospective randomised trials are needed to evaluate the long-term effects of RBC transfusion in critically ill patients.

## Supplementary Information

**Additional file 1:** Figures and Tables.

**Additional file 2:** Supplemental Text.

## Data Availability

AM had full access to all data in the study and take responsibility for the integrity of the data and the accuracy of the data analysis. The statistical code used to perform these analyses is provided with a random sample of 200 patients from the FROG-ICU database at https://osf.io/dr8gy/.

## Abbreviations

AIPTW-AIPCW: Augmented inverse probability of treatment weighting—augmented inverse probability of censoring weighting
eGFR: Estimated glomerular filtration rate
FROG-ICU: French and European outcome registry in intensive care units
Hb: Hemoglobin
HR: Hazard ratio
ICU: Intensive care unit
IL-6: Interleukin 6
IPTW: Inverse probability of treatment weighting
IQR: Inter-quartile range
L-FABP: Liver fatty acid binding protein
MIA: Missingness incorporated in attributes
MICE: Multiple imputations by chained equations
NGAL: Neutrophil gelatinase associated lipocalin
NT-proBNP: N-terminal pro-Brain natriuretic peptide
penKid: Proenkephalin A 119–159
RBC: Red blood cells
RMST: Restricted mean survival times
SAPS: Simplified acute physiology score
SD: Standard deviation
TG: Transfusion group
