# Supplementary material for: Association between in-ICU red blood cells transfusion and 1-year mortality in ICU survivors
Source: Crit Care. 2022 Oct 7;26:307. doi: 10.1186/s13054-022-04171-1 (PMC9547456; doi:10.1186/s13054-022-04171-1)
Supplement: Supplementary file 1 — Additional file 1: Figures and Tables. [file 13054_2022_4171_MOESM1_ESM.docx]

**Additional File 1 Figures and Tables**

**Association between in-ICU red blood cells transfusion and one-year mortality in ICU survivors**

Table of contents

[1. Supplemental Figures 3](#_Toc115101035)

[Figure S1: Summary of the statistical analysis performed. MICE: multiple imputations by chained equations RF-MIA: random forest-missingness incorporated in attributes, AIPTW-AIPCW: augmented inverse probability of treatment weighting - augmented inverse probability of censoring weighting, IPTW: inverse probability of treatment weighting. 3](#_Toc115101036)

[Figure S2: Absolute number of missing values in patients discharged alive in the FROG ICU cohort (n=1551). A: admission, CRP: C reactive protein, CV: cardiovascular, D: discharge, DBP: diastolic blood pressure, eGFR: glomerular filtration rate, I: inclusion, IL6: interleukin 6, LFABP: liver fatty acid binding protein (log), NGAL: neutrophil gelatinase associated lipocalin, p: plasma, PT: prothrombin time, RBC: red blood cells, RRT: renal replacement therapy, SBP: systolic blood pressure. 4](#_Toc115101037)

[Figure S3: Directed acyclic diagram for variables associated with both the transfusion and the one-year mortality (red) and variables only associated with the outcome (blue). A, admission; I, Inclusion, A-I, Admission – Inclusion. DBP: diastolic blood pressure, eGFR: estimated glomerular filtration rate, RRT: renal replacement therapy, SAPS2: simplified acute physiology score II, SBP: systolic blood pressure, SOFA: sequential organ failure assessment 5](#_Toc115101038)

[Variables are listed in the below table 5](#_Toc115101039)

[Figure S4: Flow chart. 7](#_Toc115101040)

[Figure S5: Timing of the first RBC transfusion from inclusion in the FROG-ICU cohort. Patients in whom the first transfusion was administered after day 25 are not represented on the Figure (n= 4 patients) 8](#_Toc115101041)

[Figure S6: Time-course of lowest hemoglobin values from admission to discharge in the FROG-ICU cohort according to transfusion status. Data were collected at admission, day 1 to 3, and then twice a week until discharge. The p value represents the interaction between time points and transfusion status on hemoglobin values. The dashed line represents the recommended threshold of transfusion. 9](#_Toc115101042)

[Figure S7: Probability to receive transfusion in the transfusion group and in the non-transfusion group in the FROG-ICU cohort. Panel A: The probability has been estimated according to **a logistic regression** with the baseline confounding variables excluding those impacting only the outcome (blue covariates of Figure E2). Panel B: The estimation of the probability has been done using **random forests** with all the confounding variables excluding those impacting only the outcome (blue covariates of Figure E2). 10](#_Toc115101043)

[Figure S8: Standardized mean difference of confounding variables for the unweighted population and the weighted population given the inverse probability weighting score in the FROG-ICU cohort. Confounding variables included variables at baseline associated to both transfusion prescription and outcome. I: inclusion; neuro: neurological failure; ARF: acute respiratory failure, PO: post-operative scheduled and unscheduled; CS: cardiogenic shock and cardiac arrest; SS: severe sepsis or septic shock; HS: hemorrhagic shock including trauma; others: acute liver failure, acute kidney failure, hypovolemic shock, anaphylactic shock, multiple organ failure, acute pancreatitis, metabolic; F: female; cv: cardiovascular, C-V: cardio-vascular, eGFR: glomerular filtration rate; PT: prothrombine time. 11](#_Toc115101044)

[Figure S9: Sensitivity analysis for clinical variables according to parameters at discharge in the unweighted FROG-ICU cohort. Reference is no transfusion* continuous variables were dichotomized according to median. 13](#_Toc115101045)

[Figure S10: Sensitivity analysis for biomarkers variables according to parameters at discharge in the unweighted FROG-ICU cohort. Reference is no transfusion* continuous variables were dichotomized according to median. 14](#_Toc115101046)

[Figure S11: Haptoglobin level normalized by IL-6 level in the unweighted FROG-ICU cohort. panel A: in RBC transfusion vs RBC no transfusion, panel B: according to eGFR ranges (parametric imputation with MICE). 15](#_Toc115101047)

[Figure S12: Hazard ratio of death according to the number of packed RBC administered during ICU stay in the unweighted FROG-ICU cohort. 16](#_Toc115101048)

[Supplemental Tables 17](#_Toc115101049)

[Table S1: Exploratory analyses in the unweighted FROG-ICU cohort: Renal biomarkers (log transformed) according to RBC transfusion during ICU stay. eGFR: glomerular filtration rate, NGAL: neutrophil gelatinase associated lipocalin, p: plasma, u: urinary. 17](#_Toc115101050)

[Table S2: Exploratory analyses in the unweighted FROG-ICU cohort: Cardiac biomarkers (log transformed) according to RBC transfusion during ICU stay. 18](#_Toc115101051)

## Supplemental Figures

### Figure S1: Summary of the statistical analysis performed. MICE: multiple imputations by chained equations RF-MIA: random forest-missingness incorporated in attributes, AIPTW-AIPCW: augmented inverse probability of treatment weighting - augmented inverse probability of censoring weighting, IPTW: inverse probability of treatment weighting.

### Figure S2: Absolute number of missing values in patients discharged alive in the FROG ICU cohort (n=1551). A: admission, CRP: C reactive protein, CV: cardiovascular, D: discharge, DBP: diastolic blood pressure, eGFR: glomerular filtration rate, I: inclusion, IL6: interleukin 6, LFABP: liver fatty acid binding protein (log), NGAL: neutrophil gelatinase associated lipocalin, p: plasma, PT: prothrombin time, RBC: red blood cells, RRT: renal replacement therapy, SBP: systolic blood pressure.


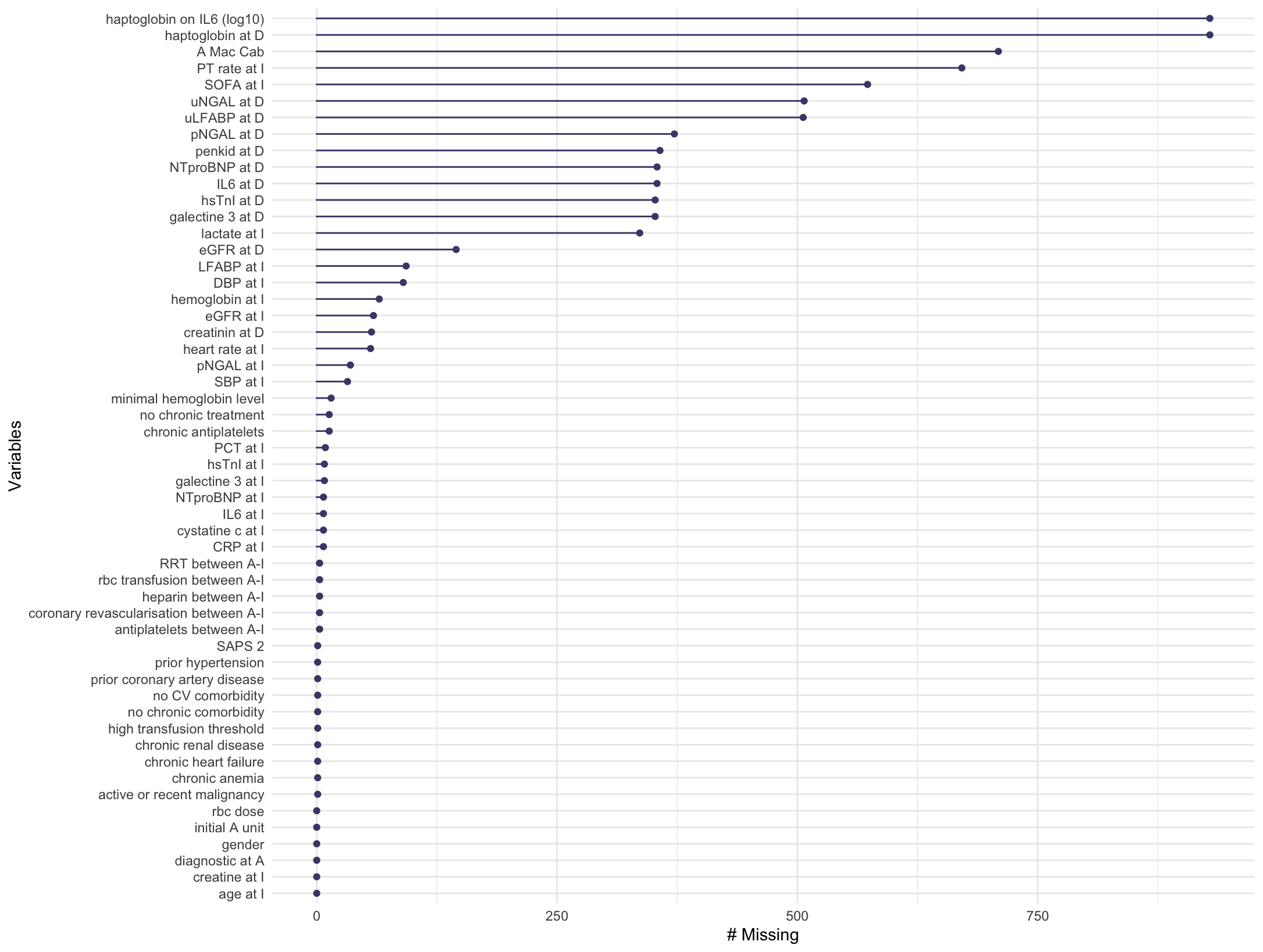


### Figure S3: Directed acyclic diagram for variables associated with both the transfusion and the one-year mortality (red) and variables only associated with the outcome (blue). A, admission; I, Inclusion, A-I, Admission – Inclusion. DBP: diastolic blood pressure, eGFR: estimated glomerular filtration rate, RRT: renal replacement therapy, SAPS2: simplified acute physiology score II, SBP: systolic blood pressure, SOFA: sequential organ failure assessment

### Variables are listed in the below table

| **Variables associated to both transfusion decision and outcome** | **Variables associated to the outcome alone** |
| --- | --- |
| *Demographic* | *Biomarkers* |
| Gender | CRP at I |
| Age at A | Galectine 3 at I |
| *Medical history* | PCT at I |
| Mac Cabe Score | hsTnI at A |
| Chronic heart failure | LFABP at I |
| Prior hypertension | cystatine c at I |
| Prior coronary artery disease | pNGAL at I |
| No cardiovascular comorbidity | IL6 at I |
| Chronic renal disease | NTproBNP at I |
| Chronic anemia |  |
| Active or recent malignancy |  |
| No chronic comorbidity |  |
| *Chronic treatments* |  |
| Chronic antiplatelets |  |
| No chronic treatment |  |
| *Management between A and I* |  |
| RBC transfusion between A-I |  |
| RRT between A-I |  |
| Coronary revascularisation between A-I |  |
| Heparin between A-I |  |
| Antiplatelets between A-I |  |
| Initial A unit |  |
| *Clinical presentation at I* |  |
| Diagnostic at I |  |
| High transfusion threshold |  |
| SAPS2 |  |
| SOFA at I |  |
| Hemoglobin at I |  |
| SBP at I |  |
| DBP at I |  |
| Heart rate at I |  |
| eGFR at I |  |
| Lactate at I |  |
| Creatinin at I |  |
| PT rate at I |  |

### Figure S4: Flow chart.

### Figure S5: Timing of the first RBC transfusion from inclusion in the FROG-ICU cohort. Patients in whom the first transfusion was administered after day 25 are not represented on the Figure (n= 4 patients)

### Figure S6: Time-course of lowest hemoglobin values from admission to discharge in the FROG-ICU cohort according to transfusion status. Data were collected at admission, day 1 to 3, and then twice a week until discharge. The p value represents the interaction between time points and transfusion status on hemoglobin values. The dashed line represents the recommended threshold of transfusion.

### Figure S7: Probability to receive transfusion in the transfusion group and in the non-transfusion group in the FROG-ICU cohort. Panel A: The probability has been estimated according to **a logistic regression** with the baseline confounding variables excluding those impacting only the outcome (blue covariates of Figure E2). Panel B: The estimation of the probability has been done using **random forests** with all the confounding variables excluding those impacting only the outcome (blue covariates of Figure E2).


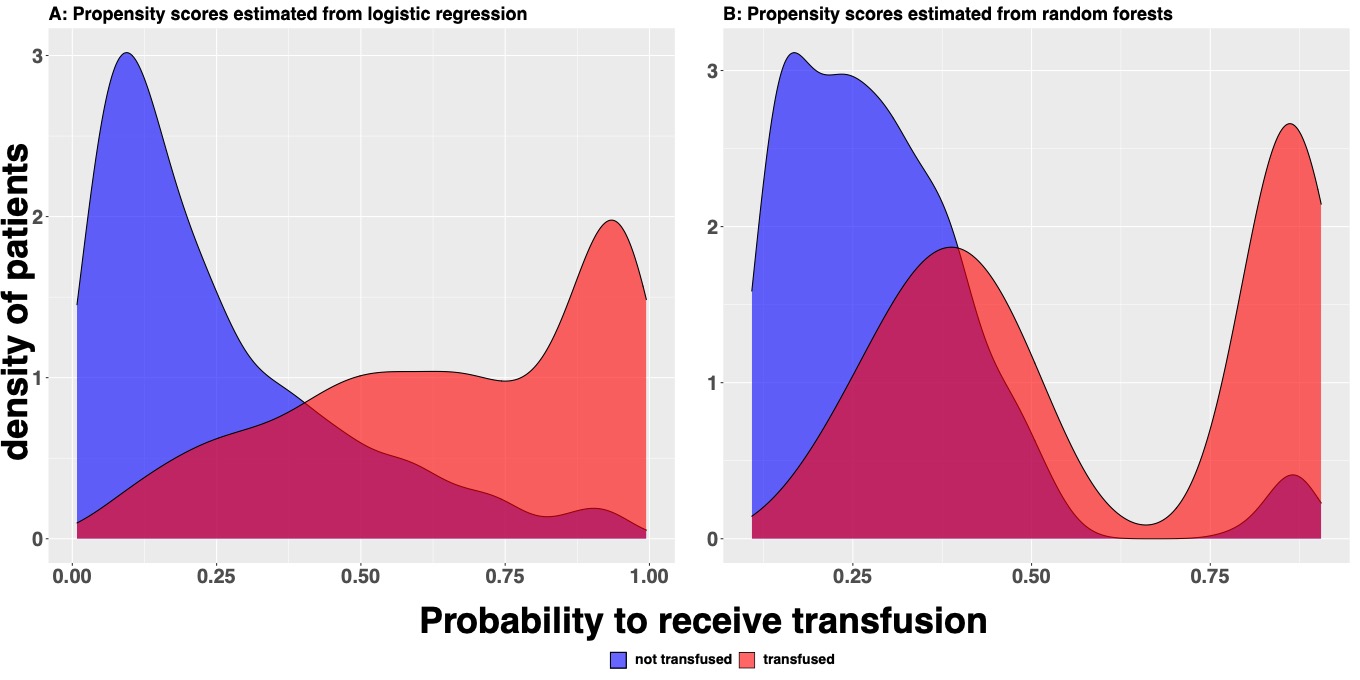


### Figure S8: Standardized mean difference of confounding variables for the unweighted population and the weighted population given the inverse probability weighting score in the FROG-ICU cohort. Confounding variables included variables at baseline associated to both transfusion prescription and outcome. I: inclusion; neuro: neurological failure; ARF: acute respiratory failure, PO: post-operative scheduled and unscheduled; CS: cardiogenic shock and cardiac arrest; SS: severe sepsis or septic shock; HS: hemorrhagic shock including trauma; others: acute liver failure, acute kidney failure, hypovolemic shock, anaphylactic shock, multiple organ failure, acute pancreatitis, metabolic; F: female; cv: cardiovascular, C-V: cardio-vascular, eGFR: glomerular filtration rate; PT: prothrombine time.

### Figure S9: Sensitivity analysis for clinical variables according to parameters at discharge in the unweighted FROG-ICU cohort. Reference is no transfusion* continuous variables were dichotomized according to median.

### Figure S10: Sensitivity analysis for biomarkers variables according to parameters at discharge in the unweighted FROG-ICU cohort. Reference is no transfusion* continuous variables were dichotomized according to median.

### Figure S11: Haptoglobin level normalized by IL-6 level in the unweighted FROG-ICU cohort. panel A: in RBC transfusion vs RBC no transfusion, panel B: according to eGFR ranges (parametric imputation with MICE).

### Figure S12: Hazard ratio of death according to the number of packed RBC administered during ICU stay in the unweighted FROG-ICU cohort.


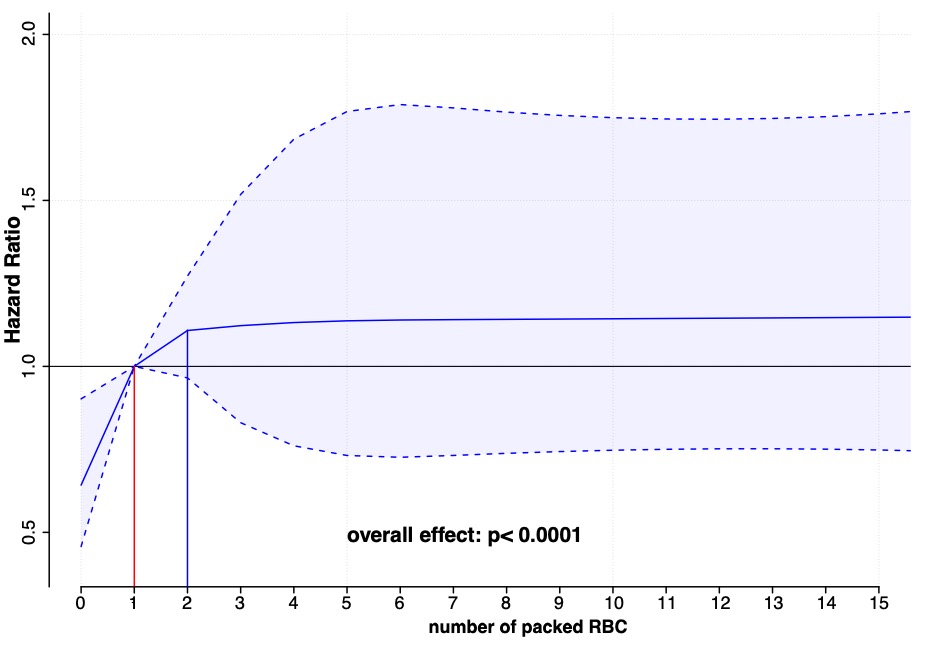


## Supplemental Tables

### Table S1: Exploratory analyses in the unweighted FROG-ICU cohort: Renal biomarkers (log transformed) according to RBC transfusion during ICU stay. eGFR: glomerular filtration rate, NGAL: neutrophil gelatinase associated lipocalin, p: plasma, u: urinary.

| **Variables at discharge** | **Global mean±SD^*^** | **No transfusion  mean±SD^*^** | **Transfusion  mean±SD^*^** | **p-value** |
| --- | --- | --- | --- | --- |
| eGFR (log) | 4.47 ± 0.75 | 4.56 ± 0.65 | 4.36 ± 0.84 | <0.0001 |
| Creatinine (log) | 4.33 ± 0.66 | 4.26 ± 0.59 | 4.43 ± 0.73 | <0.0001 |
| p NGAL (log) | 4.75 ± 1.28 | 4.55 ± 1.27 | 5.03 ± 1.25 | <0.0001 |
| u NGAL (log) | 4.23 ± 1.52 | 3.99 ± 1.42 | 4.56 ± 1.59 | <0.0001 |
| u liver fatty acid binding protein (log) | 4.24 ± 0.73 | 4.1 ± 0.63 | 4.43 ± 0.82 | <0.0001 |
| p PenKid (log) | 2.67 ± 1.39 | 2.41 ± 1.3 | 3.03 ± 1.42 | <0.0001 |
| ^*^imputed dataset was used. | | | | |

### Table S2: Exploratory analyses in the unweighted FROG-ICU cohort: Cardiac biomarkers (log transformed) according to RBC transfusion during ICU stay.

| **Variables at discharge** | **Global mean±SD^*^** | **No transfusion  mean±SD^*^** | **Transfusion  mean±SD^*^** | **p value** |
| --- | --- | --- | --- | --- |
| Galectin 3 (log) | 3.02 ± 0.53 | 2.91 ± 0.48 | 3.16 ± 0.56 | <0.0001 |
| NT pro-BNP (log) | 6.36 ± 1.92 | 6.09 ± 1.92 | 6.73 ± 1.86 | <0.0001 |
| hs Troponin I (log) | 2.75 ± 1.85 | 2.69 ± 1.91 | 2.83 ± 1.77 | 0.168 |
| ^*^imputed dataset was used | | | | |
