# Supplementary material for: Association between in-ICU red blood cells transfusion and 1-year mortality in ICU survivors
Source: Crit Care. 2022 Oct 7;26:307. doi: 10.1186/s13054-022-04171-1 (PMC9547456; doi:10.1186/s13054-022-04171-1)
Supplement: Supplementary file 2 — Additional file 2: Supplemental Text. [file 13054_2022_4171_MOESM2_ESM.docx]

**Additional file 2 Supplemental Text**

**Association between in-ICU red blood cells transfusion and one-year mortality in ICU survivors**

Table of contents

[1. Statistical analysis 1](#_Toc103004305)

[1.1. Missing data management 1](#_Toc103004306)

[1.2. Causal inference models 2](#_Toc103004307)

[1.2.1. Parametric and non-parametric approaches of the different models 2](#_Toc103004308)

[1.2.2. Estimators 3](#_Toc103004309)

[1.2.2.1. the crude estimation with no adjustment 3](#_Toc103004310)

[1.2.2.2. the inverse probability of treatment weighting (IPTW) with the Kaplan Meier estimator 3](#_Toc103004311)

[1.2.2.3. the doubly robust estimator- AIPTW-AIPCW 3](#_Toc103004312)

[2. References 4](#_Toc103004313)

### Statistical analysis

#### Missing data management

We have used two approaches for handling the missing values. A parametric imputation method: Multiple imputation by chained equations (MICE)[1] and a non-parametric method without imputation: Random forests-Missingness incorporated in attributes (MIA) [2].

- - 1. *Parametric imputation method*

Two multiple imputations were performed to replace missing values when appropriate:

a) For the estimation of the effect of the transfusion, 15 imputed datasets were generated from the initial dataset for baseline characteristics only.

b) For the explanatory analysis, 15 imputed datasets were generated from the initial dataset for baseline and discharge characteristics.

Analyses were performed on each dataset and the results were pooled into a final result using Rubin’s rule [3]. When the missing covariates were imputed with multiple imputations, the confidence interval were obtained following the methodology Boot MI of Schomaker et al [4].

- - 1. *Non-parametric method without imputation*

Random forests-Missingness incorporated in attributes (MIA). The missing data were managed by MIA in the random forests methods which does not impute the missing data but integrated it in the tree criteria. For each split made in the decision’s trees, the missing value was considered as a category in the decision. Therefore, the missing value was either assigned to the right or the left node of the split [2].

In the first parametric approach of the different models (censoring function, survival function, propensity score) missing data were imputed with MICE and in the second non-parametric approach of the different models (censoring function, survival function, propensity score) missing data were handled with MIA.

#### Causal inference models

##### Parametric and non-parametric approaches of the different models

We have considered two different ways to estimates these models: one semi-parametric approach and one non-parametric approach.

In the first semi-parametric approach, we used cox models for modelling the survival and censoring functions and a logistic regression for modelling the propensity score. We have used for these two first models all red and blue covariates (Figure S3) while we have only used the blue covariates for the estimation of the propensity score. Red covariates are associated with both transfusion and outcome (one-year mortality) whereas blue covariates are only associated with the outcome.

In the second non-parametric approach, we modelled the survival function and the censoring probability with random survival forest (RSF) with all red and blue covariates. We modelled the propensity score from random forests (RF) with only the blue covariates. This non-parametric method is not constrained by linear hypothesis thus allowing to consider the complex relationship between variables and interaction [5]. Briefly, the “forest” is an ensemble of trees created from bootstrapped datasets and then aggregated (bagging method) to obtain a more accurate prediction.

##### Estimators

###### the crude estimation with no adjustment

A Kaplan Meier estimator was used separately for patients with and without transfusion. This method was used for comparison purposes [6].

###### the inverse probability of treatment weighting (IPTW) with the Kaplan Meier estimator

For IPTW adjustment, each patient was weighted using the inverse of the estimated propensity score. And then similarly to the latter method, a Kaplan Meier estimator was used separately for patients with and without transfusion. This method is adjusted for confounding bias but not for censoring. Only blue covariates were used in the model.

###### the doubly robust estimator- AIPTW-AIPCW

We combined an outcome regression model (i.e., G-formula) with both a model for the treatment (i.e., IPTW) and a model for the probability of remaining uncensored. This combination resulted in an augmented inverse probability weighting (AIPTW-AIPCW) estimation which is a more effective approach in minimizing biases due to robustness to model misspecification compared to only inversed probability of weighting estimators (IPTW-IPCW) or the G-formula alone [7]. In the non-parametric approach, an additional step in this estimator is necessary to preserve consistency, namely cross-fitting as pointed out by Ozenne et al [7]. For this, the doubly robust estimator from Cui et al. was used [8].

#### References

1. van Buuren S, Groothuis-Oudshoorn K: **mice: Multivariate Imputation by Chained Equations in R**. *Journal of Statistical Software* 2011, **45**(3):1 - 67.

2. Twala BETH, Jones MC, Hand DJ: **Good methods for coping with missing data in decision trees**. *Pattern Recogn Lett* 2008, **29**(7):950–956.

3. Rubin DB: **Inference and Missing Data**. *Biometrika* 1976, **63**(3):581–592.

4. Schomaker M, Heumann C: **Bootstrap inference when using multiple imputation**. *Stat Med* 2018, **37**(14):2252-2266.

5. Ishwaran H, Kogalur UB, Blackstone EH, Lauer MS: **Random survival forests**. *The Annals of Applied Statistics* 2008, **2**(3):841-860, 820.

6. Kaplan EL, Meier P: **Nonparametric Estimation from Incomplete Observations**. *Journal of the American Statistical Association* 1958, **53**(282):457–481.

7. Ozenne BMH, Scheike TH, Staerk L, Gerds TA: **On the estimation of average treatment effects with right-censored time to event outcome and competing risks**. *Biom J* 2020, **62**(3):751-763.

8. Cui Y, Kosorok MR, Sverdrup E, Wager S, R. Z: **Estimating heterogeneous treatment effects with right-censored data via causal survival forests.** . *arXiv preprint arXiv* 2001.09887 2020.
